# Neuroendoscopic surgery for septated chronic subdural hematoma

**DOI:** 10.3389/fsurg.2025.1703624

**Published:** 2026-01-07

**Authors:** Shelin Liu, Fei Liu, Zheng Cai, Xudong Zhao

**Affiliations:** 1 Department of Neurosurgery, Wuxi No. 2 People’s Hospital, Affiliated Wuxi Clinical College of Nantong University, Wuxi, China; 2 Department of Neurosurgery, Wuxi No. 2 People’s Hospital, Wuxi, China; 3 Department of Neurosurgery, Jiangnan University Medical Center, Wuxi, China

**Keywords:** neuroendoscopic, prognosis, recurrence, septated chronic subdural hematomas, surgery

## Abstract

**Objective:**

To explore the clinical efficacy of neuroendoscopic surgery in the treatment of septated chronic subdural hematomas (sCSDH).

**Methods:**

A retrospective analysis was conducted on the clinical data of 97 patients with sCSDH admitted to the Department of Neurosurgery, Wuxi Second People's Hospital from June 2019 to March 2025. Among them, patients who underwent traditional burr hole drainage under local anesthesia were assigned to the traditional burr hole drainage group (control group, *n* = 52), and those who underwent neuroendoscopic subdural hematoma evacuation under general anesthesia were assigned to the neuroendoscopic surgery group (observation group, *n* = 45). Perioperative indicators, Glasgow Coma Scale (GCS) score, Markwalder grade, neurological function scores before and after surgery, complications, and hematoma recurrence within 3 months of follow-up were compared between the two groups.

**Results:**

Before surgery, there were no significant differences in perioperative indicators, GCS score, Bender grade, or Markwalder grade between the two groups (*P* > 0.05). The observation group showed better intraoperative blood loss, drainage tube indwelling time, and immediate postoperative hematoma clearance rate than the control group (*P* < 0.05), while the total operation time of the control group was shorter than that of the observation group (*P* < 0.05). After surgery, neurological function scores improved in both groups (*P* < 0.05), but there was no significant difference between the two groups (*P* > 0.05). At different time points after surgery, GCS scores decreased in both groups (*P* < 0.05), and the scores in the observation group were lower than that in the control group (*P* < 0.05). There were no significant differences in the total complication rate or length of hospital stay between the two groups (*P* > 0.05), but the hematoma recurrence rate in the observation group was lower than that in the control group (*P* < 0.05).

**Conclusion:**

Both neuroendoscopic surgery and traditional burr hole drainage are effective in the treatment of sCSDH. However, neuroendoscopic surgery achieves a higher hematoma clearance rate, better recovery of consciousness, neurological function, and activities of daily living, and a lower postoperative recurrence rate, making it worthy of clinical promotion.

## Introduction

Chronic subdural hematoma (CSDH) is a disease caused by abnormal accumulation of blood in the subdural space, usually associated with trauma, coagulation disorders, brain atrophy, and other factors, and is more common in the elderly population (1, 2). CSDH is often accompanied by symptoms such as headache, vomiting, and disturbance of consciousness; in severe cases, it may even cause hemiplegia, aphasia, and other symptoms, threatening patients' health and affecting their normal lives (3). Due to population aging and the use of anticoagulant/antiplatelet drugs, the complication rate of CSDH is on the rise (4). Septated chronic subdural hematoma (sCSDH) is a special type of CSDH formed by the division of the hematoma cavity by multiple fibrous septa. Its pathological feature is that the septa in the hematoma cavity hinder the outflow of hematoma fluid, which easily leads to failed hematoma evacuation and a high recurrence rate (5, 6). How to efficiently remove the hematoma, improve patients' postoperative neurological function, enhance their quality of life, and reduce the hematoma recurrence rate has become a current research focus.

At present, surgery is a common treatment for sCSDH, among which burr hole drainage with a closed drainage system is the preferred surgical option due to its advantages of simplicity, safety, and cost-effectiveness, and is widely used in clinical practice (7, 8). However, burr hole drainage also has adverse reactions such as postoperative effusion, pneumocephalus, and a relatively high hematoma recurrence rate. Especially in the treatment of sCSDH patients, the presence of septa in the hematoma cavity easily leads to incomplete hematoma evacuation (9, 10). On the basis of burr hole drainage, neuroendoscopic surgery can perform hemostasis and hematoma evacuation accurately under direct vision, thereby improving the hematoma clearance rate and reducing the recurrence rate (4, 11, 12). From June 2019 to March 2025, our hospital admitted 97 patients with sCSDH, among whom 45 were treated with neuroendoscopic subdural hematoma evacuation and 52 with traditional burr hole drainage. The clinical efficacy of the two methods was compared and evaluated.

## Materials and methods

### General information

A retrospective analysis was performed on the clinical data of patients with sCSDH admitted to Wuxi Second People's Hospital from June 2019 to March 2025.

Inclusion criteria: (1) Diagnosis of two-cavity or multi-cavity sCSDH confirmed by cranial CT or MRI, with a course of disease >3 weeks; (2) Complete clinical data and no surgical contraindications; (3) Glasgow Coma Scale (GCS) score >8; (4) Hematoma thickness [maximum thickness measurement: On axial CT images, the slice showing the widest part of the hematoma on the surgical side was selected, and the maximum width of the hematoma was measured perpendicular to the inner table of the skull (13)] ≥1.5 cm, with sufficient space for rigid neuroendoscopic operation.

Exclusion criteria: (1) GCS score <5 or dilated pupils; (2) Coagulation disorders; (3) Hematoma in the acute or subacute phase.

**Figure 1:**
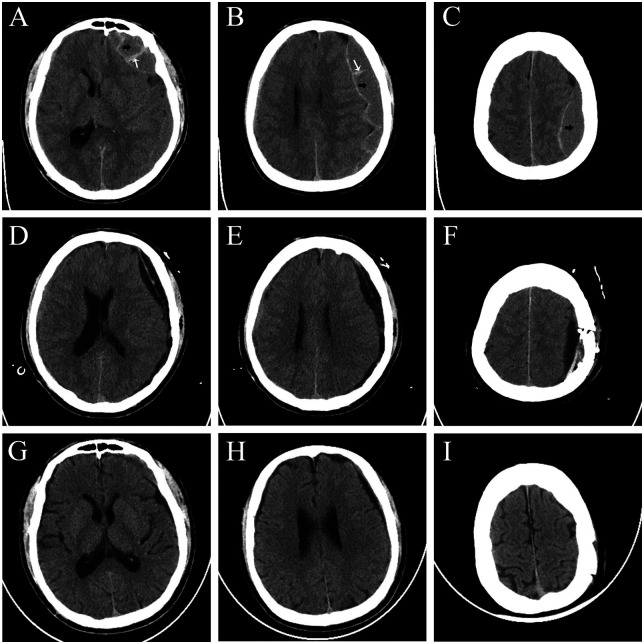
**(A–C)** sCSDH preoperative CT image, black arrow represents hematoma, white arrow represents fibrous septum inside hematoma. **(D–F)** CT imaging of the brain one week after endoscopic treatment. **(G–I)** CT imaging of the brain one month after endoscopic treatment.

**Figure 2:**
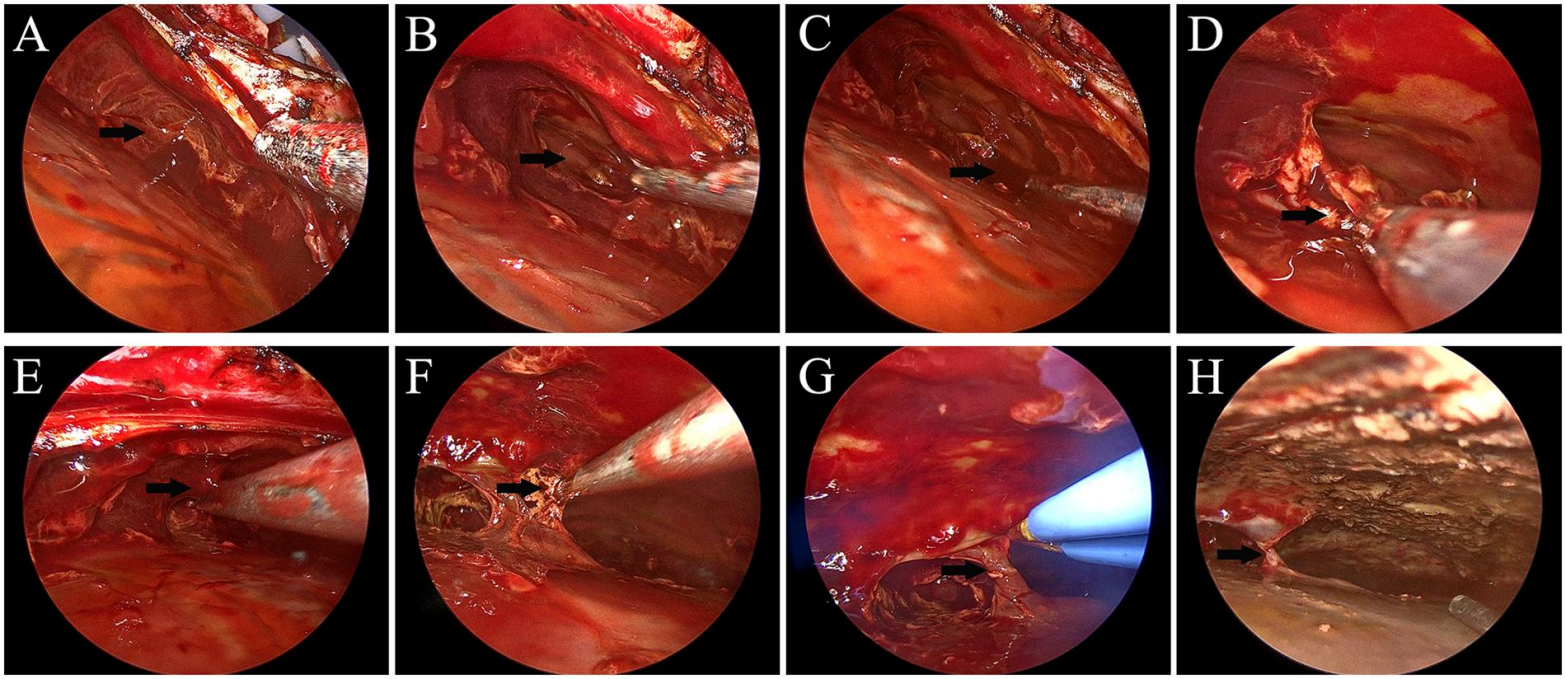
Endoscopic treatment of sCSDH. **(A)** Multiple fibrous septa within the hematoma. **(B)** Fistula creation. **(C)** Clear liquefied hematoma. **(D)** Remove hematoma separation. **(E)** Clear the organized hematoma. **(F)** Remove hematoma separation. **(G)** Hemostasis under endoscopic vision. **(H)** Conversion of multilocular hematoma to single locular hematoma.

Patients were divided into two groups according to the surgical method: the control group (treated with traditional burr hole drainage, *n* = 52) and the observation group (treated with neuroendoscopic surgery, *n* = 45). The general data of the two groups were balanced and comparable (Table 1). This study strictly followed the Helsinki Declaration and was approved by the Ethics Committee of Wuxi No.2 People's Hospital (WXEY-2025-138). Preoperative and postoperative CT images of patients are shown in Figure 1, and intraoperative neuroendoscopic treatment of sCSDH is shown in Figure 2.

**Table 1:** Comparison of preoperative general data between the two groups.

General data	Control group (*n* = 52)	Observation group (*n* = 45)	t/*χ*^2^/*Z*	*P*
Gender (*n*, male/female)	24/28	25/20	0.995	0.319
Age (years)	72.41 ± 6.92	71.83 ± 7.12	0.41	0.685
Trauma history (*n*)	21 (40.38%)	19 (42.22%)	0.019	0.891
Hypertension history (*n*)	11 (21.15%)	9 (20.00%)	0.032	0.858
Diabetes mellitus history (*n*)	5 (9.62%)	5 (11.11%)	0.136	0.713
Coronary artery disease history (*n*)	5 (9.62%)	4 (8.89%)	0.015	0.903
Cerebral infarction history (*n*)	2 (3.85%)	2 (4.44%)	0.008	0.928
Antiplatelet therapy (*n*)	3 (5.77%)	3 (6.67%)	0.012	0.913
Anticoagulant therapy (*n*)	1 (1.92%)	1 (2.22%)	0.009	0.924
Hematoma laterality (*n*, unilateral/bilateral)	40/12	33/12	0.17	0.682
Hematoma volume **(**mL**)**	110.58 ± 9.42	107 ± 10.29	1.81	0.073
Preoperative hematoma thickness (cm)	2.55 ± 0.32	2.57 ± 0.38	0.40	0.693
Preoperative midline shift (*n*)			0.606	0.739
** <**1 cm	18	16		
** **1–2 cm	22	16		
** **>2 cm	12	13		
Preoperative GCS score	13.17 ± 2.09	12.85 ± 1.94	0.84	0.404
Bender grade at admission (*n*)			0.28	0.781
** **Grade 0	0	0		
** **Grade Ⅰ	18	15		
** **Grade Ⅱ	24	21		
** **Grade Ⅲ	7	8		
** **Grade Ⅳ	3	1		

## Surgical methods

### Control group: surgery under local anesthesia

Based on preoperative imaging localization, a transverse incision of approximately 4 cm was made centered at the thickest layer of the hematoma (mostly located at the parietal tuber). The scalp was incised to expose the skull, a burr hole was drilled with a craniotome, and a 1 × 2 cm bone window was created with a milling cutter. The dura mater was cauterized with electrocoagulation, and a “+” incision was made in two layers with a sharp blade for hemostasis. Dark brown or dark red non-coagulated fluid was observed to flow out, and repeated irrigation was performed. A drainage tube was inserted, and gentle and repeated irrigation was conducted with normal saline. After satisfactory irrigation, the burr hole was filled with gelatin sponge, the drainage tube was subcutaneously tunnelled and brought out of the skin. Hemostasis was achieved thoroughly, the scalp was sutured in two layers, and the drainage tube was connected to a drainage bag.

### Observation group: surgery under general anesthesia

Based on preoperative imaging localization, a transverse incision of approximately 6 cm was made centered at the thickest layer of the hematoma (mostly located at the parietal tuber). The scalp was incised to expose the skull. A burr hole was drilled with a drill, and an elliptical bone flap of approximately 4 × 3 cm was created with a milling cutter. The edge of the bone window was filled with bone wax, and the dura mater was suspended with silk thread. The dura mater was cauterized with electrocoagulation, and a “+” incision was made in two layers with a sharp blade for hemostasis. The capsule of the parietal hematoma cavity was exposed, cauterized with electrocoagulation, and then sharply incised. A neuroendoscope (0-degree Storze rigid endoscope) was connected, and the hematoma and hematoma capsule were gently aspirated with an aspirator under direct vision. Fibrous septa were removed, and hemostasis was achieved with bipolar electrocoagulation. Repeated and gentle irrigation was performed with normal saline. After satisfactory irrigation, a drainage tube was inserted into the occipital region, the burr hole was filled with gelatin sponge, and the drainage tube was subcutaneously tunnelled and brought out of the skin. The dura mater was sutured tightly, the skull was repaired with two connecting plates, the scalp was sutured in two layers, and the drainage tube was connected to a drainage bag.

### Postoperative management

All patients were placed in a head-down and foot-up position after surgery and resumed eating on the next day. They were given oral atorvastatin calcium tablets 20 mg/d routinely, and fluid infusion was about 2,000 mL/d. The dose was appropriately reduced for elderly patients with cardiac insufficiency. Cranial CT was rechecked on the day after surgery, and the indwelling time of the drainage tube was determined according to the residual hematoma volume. It was imperative to maintain unobstructed drainage and monitor the volume and color of the drainage fluid. The drainage tube was generally removed within 1–3 days. Cranial CT was rechecked daily before tube removal, and again on the day after tube removal and at discharge. Follow-up cranial CT was performed 2 weeks after discharge, and then at 1, 3, 6, and 12 months after discharge.

### Observation indicators

(1) Surgery-related indicators: The differences in operation time (min), intraoperative blood loss (mL), drainage tube indwelling time (h), and length of hospital stay (d) between the two groups were statistically analyzed. The immediate postoperative hematoma clearance rate was calculated as follows: Clearance rate = (Preoperative hematoma volume—Postoperative hematoma volume)/Preoperative hematoma volume × 100%.
(2) Coma status: The Glasgow Coma Scale (GCS) was used to assess the coma status of the two groups before surgery and 7 days after surgery, including three components: eyes opening (1–4 points), verbal response (1–5 points), and motor response (1–6 points). A lower score indicated more severe disturbance of consciousness.
(3) Markwalder neurological function grade: Grade 0, normal neurological function; Grade Ⅰ, clear consciousness, mild headache, normal muscle tone; Grade Ⅱ, local neurological impairment, disorientation; Grade Ⅲ, severe neurological impairment, shallow coma; Grade Ⅳ, deep coma, no response to pain, decerebrate rigidity.
(4) Complications and hematoma recurrence rate: The occurrence of complications and hematoma recurrence rate were recorded in both groups.

### Statistical analysis

GraphPad Prism 8 software was used for statistical analysis. Paired sample t-test was used for intragroup comparison, independent sample *t*-test for intergroup comparison, analysis of variance for multiple groups. Count data were expressed as [*n* (%)], and intergroup comparison was performed using independent sample *χ*^2^ test. Ranked data were analyzed using nonparametric rank-sum test. A *P* value <0.05 was considered statistically significant.

## Results

### Comparison of surgery-related indicators between the two groups

The operation time and hospitalization time of the control group were significantly shorter than those of the observation group (*P* < 0.05, Table 2); The immediate hematoma clearance rate in the observation group was better than that in the control group (*P* < 0.05, Table 2), and there was no significant difference in intraoperative bleeding volume and drainage tube retention time between the two groups (*P* > 0.05, Table 2).

**Table 2:** Comparison of surgery-related indicators between the two groups (x¯ ± s).

Group	**O**peration time **(**min**)**	**I**ntraoperative blood loss **(**mL**)**	**R**etention time of drainage tube (h)	**L**ength of hospital stay (d)	**I**mmediate hematoma clearance rate after surgery (%)
Control group (*n* = 52)	81.15 ± 25.00	38.46 ± 10.55	48.23 ± 12.90	11.75 ± 1.23	74.79 ± 5.42
Observation group (*n* = 45)	117.67 ± 40.85	43.89 ± 15.95	48.80 ± 13.87	12.58 ± 1.01	90.11 ± 3.34
t	5.04	1.90	0.20	3.86	16.89
*P*	0.000	0.06	0.84	0.000	0.000

### Comparison of GCS scores between the two groups

There was no significant difference in preoperative GCS scores between the two groups of patients (*P* > 0.05, Table 3); In terms of eyes opening and movement dimensions, there was no significant difference in GCS scores between the two groups 7 days after surgery (*P* > 0.05, Table 3), while the language score observation group showed a significant improvement compared to the control group (*P* < 0.05, Table 3). In terms of eyes opening, language, and exercise, both groups showed significant improvement in GCS scores 7 days after surgery compared to preoperative levels (*P* < 0.05, Table 3).

**Table 3:** Comparison of GCS scores between the two groups (x¯ ± s).

Group	Eyes open		Linguistic ability		Movement	
	Preoperative	Postoperative 7-day	Preoperative	Postoperative 7-day	Preoperative	Postoperative 7-day
Control group (*n* = 52)	2.98 ± 0.58	3.94 ± 0.24	4.06 ± 0.61	4.81 ± 0.53	5.38 ± 0.49	5.94 ± 0.24
Observation group (*n* = 45)	3.18 ± 0.49	3.93 ± 0.25	4.24 ± 0.61	4.98 ± 0.15	5.20 ± 0.50	5.98 ± 0.15
t	1.89^a^	0.20^a^, 11.13^b^, 9.23^c^	1.48^a^	2.05^a^, 6.43^b^, 9.38^c^	1.76^a^	0.93^a^, 7.68^b^, 10.07^c^
*P*	0.06^a^	0.84^a^, 0.000^b^, 0.000^c^	0.14^a^	0.045^a^, 0.000^b^, 0.000^c^	0.082^a^	0.355^a^, 0.000^b^, 0.000^c^

a Comparison between the control group and the observation group at the same time point.

b Compare the postoperative 7-day and preoperative in control group.

c Compare the postoperative 7-day and preoperative in the observation group.

### Comparison of Markwalder neurological function grading between the two groups

Before surgery, there was no statistically significant difference in neurological function grading between the two groups (*P* > 0.05, Table 4). At 7 days after surgery, the Markwalder neurological function grading of both groups improved (*P* < 0.05, Table 4), but there was no statistically significant difference between the groups (*P* > 0.05, Table 4).

**Table 4:** Comparison of markwalder neurological function grading between the Two groups.

Group	Grade 0	Grade Ⅰ	Grade Ⅱ	Grade Ⅲ	*Z*	*P*
Control group (*n* = 52)					6.89	0.000
Preoperative	0	10	12	30		
Postoperative 7-day	25	14	12	1		
Observation group (*n* = 45)					7.21	0.000
Preoperative	0	8	11	26		
Postoperative 7-day	26	10	9	0		
*Z*	1.03					
*P*	0.303					

### Comparison of complication and hematoma recurrence rates between the two groups

The total complication rate of the observation group was lower than that of the control group, but the difference between the two groups was not statistically significant (*χ*^2^ = 0.47, *P* = 0.493, Table 5); the hematoma recurrence rate of the observation group was lower than that of the control group (*χ*^2^ = 5.18, *P* = 0.031, Table 5).

**Table 5:** Comparison of complications and recurrence rates between the Two groups [*n* (%)].

Group	Intracranial infection	Subdural hematoma	Subdural effusion	Pneumocephalus	Total complications	Hematoma recurrence
Control group (*n* = 52)	1	2	1	1	5 (9.61)	8 (15.38)
Observation group (*n* = 45)	0	1	1	1	3 (6.67)	1 (2.22)

## Discussion

### Pathogenesis of sCSDH

Chronic subdural hematoma (CSDH) is a common disease in neurosurgery, which mainly affects middle-aged and elderly people (2). CSDH consists of an outer membrane, a hematoma cavity, and an inner membrane. The inner and outer capsules change over time, and the old blood in the hematoma cavity gradually transforms into non-coagulated blood, semi-solid, or solid state, with organized fibrous tissue and septa forming, thus developing into septated chronic subdural hematoma (sCSDH). sCSDH-induced craniocerebral trauma causes a series of symptoms such as headache, nausea, vomiting, limb dysfunction, or sudden coma. Some literatures suggest that the bleeding source of sCSDH is the bridging veins extending from the cerebral cortex to the dural veins (6, 14). In elderly patients, brain atrophy increases vascular tension, leading to rupture and bleeding of bridging veins. The bleeding is not easy to coagulate spontaneously; after red blood cell rupture, metabolic products such as iron and calcium trigger a cascade reaction with bridging veins and arachnoid, resulting in repeated fibrinolysis, inflammation, coagulation, neovascularization, and capsule formation, which transforms single-chamber hematoma into multi-chamber hematoma. This is an important pathological mechanism for the occurrence and development of sCSDH (15). Based on the current mainstream hypotheses regarding the pathogenesis of CSDH, the parietal capsule contains immature microvessels with increased permeability. Blood extravasates into the hematoma cavity through these microvessels, and anticoagulant or antiplatelet therapy can exacerbate such extravasation, thereby potentially increasing the postoperative recurrence rate (16).

### Key points of sCSDH treatment

In terms of clinical management, combined with the pathogenesis and characteristics of sCSDH, the treatment of sCSDH should prioritize the thorough opening or complete evacuation of intrahematomal septa, thereby avoiding the retention of relatively isolated compartments that cannot be adequately irrigated and drained. This strategy is of great significance for improving surgical outcomes.

The key points of sCSDH treatment are: (1) Remove the hematoma and flush the hematoma cavity to reduce the intracavitary osmotic pressure; (2) Remove the septa to convert multi-chamber hematoma into single-chamber hematoma; (3) Remove the inner membrane to expose the arachnoid, promote cavity fluid absorption, and accelerate cerebral re-expansion.

### Advantages and disadvantages of burr hole drainage for sCSDH

Conservative treatment may be considered for small or moderate sCSDH. However, for patients with large hematoma volume, obvious midline shift, and obvious craniocerebral trauma symptoms, twist-drill trepanation is the conventional treatment for sCSDH, which can quickly and effectively relieve compression. However, burr hole drainage is prone to poor catheter placement, brain tissue damage, and poor drainage. Especially for sCSDH, the presence of septa leads to incomplete hematoma removal and insufficient drainage, secondary acute bleeding, which significantly increases the surgical risk and hematoma recurrence rate in elderly patients (17). Therefore, there is an urgent need for more efficient and safe treatment methods in clinical practice.

### Advantages and disadvantages of neuroendoscopic treatment for sCSDH

In recent years, with the wide clinical application of neuroendoscopy, minimally invasive treatment of sCSDH has been widely reported and achieved good results in clinical practice (18). Although this technology has been recognized, its application in the treatment of chronic subdural hematoma is not widespread. Neuroendoscopy is commonly used in the treatment of hydrocephalus, intraventricular cysts, arachnoid cysts, fourth ventricle obstruction, and other diseases, and can also be applied in other spinal and brain surgeries. The results of this study showed that: the intracranial hematoma clearance rate of the observation group was higher than that of the control group; the indwelling time of the drainage tube in the hematoma cavity, complication rate, and hematoma recurrence rate of the observation group were lower than those of the control group (*P* < 0.05); the Markwalder neurological function grading of the observation group was better than that of the control group (*P* < 0.05); the postoperative hospital stay of the control group was lower than that of the observation group.

The advantages of neuroendoscopic sCSDH removal are as follows: the operation is performed under general anesthesia, which is not affected by the patient's own factors, avoiding the disadvantages of patient restlessness and poor compliance under local anesthesia; it can thoroughly flush the hematoma cavity and obtain a good catheter placement position; neuroendoscopy is operated under direct vision with clear anatomical field, enabling more effective treatment of the hematoma cavity and inner membrane, accurate aspiration of blood clots and fibrotic tissue, precise electrocoagulation of bleeding points, and opening and resection of septal capsules (19). For blood clots that cannot be removed at the edge, a large amount of normal saline can be used for repeated targeted flushing, effectively preventing bleeding and hematoma recurrence. Intraoperative surgical trauma is reduced, surgical risk is lowered, postoperative recurrence rate is effectively reduced, complications are decreased, and hospital stay is effectively shortened (20). Although studies have reported the completion of neuroendoscopic hematoma evacuation under local anesthesia, this approach carries a relatively high risk. Specifically, it is challenging for patients to achieve absolute cooperation and maintain a stable head position intraoperatively, which may easily lead to injuries to brain tissue, blood vessels, and other structures, thereby resulting in more severe complications (21). On the other hand, general anesthesia is not always feasible (especially in elderly patients with multiple comorbidities) and may increase the risk of severe postoperative complications associated with endotracheal intubation and mechanical ventilation support (22, 23). Therefore, the choice between general and local anesthesia should be made based on a comprehensive preoperative assessment to reach a decision that maximizes benefits for the patient. Upon reviewing the cases in this study, we observed that complications occurred infrequently in both patient groups postoperatively. This finding indicates that the surgical approaches employed in the study are free of major flaws and adhere to safety and standardization principles.

However, neuroendoscopic treatment of sCSDH may have the following problems: first, the endoscope is limited by the bone window, unable to reach the edge of the hematoma, making it difficult to observe the distally curved hematoma cavity and fully remove the hematoma in this area; second, after partial hematoma removal, the brain tissue may bulge rapidly, leading to “occlusion and surround” of the distal hematoma cavity, making it difficult for the endoscope to reach the distal hematoma cavity (2). Therefore, in this study, we changed the conventional straight incision to a transverse incision, making the long axis of the bone window consistent with the long axis of the hematoma. This not only avoids the obstruction of the endoscope by the mastoid retractor but also greatly increases the range of motion of the rigid endoscope, ensuring that the endoscope can reach the edge of the hematoma and adjust the order of hematoma removal, thereby removing the hematoma as thoroughly as possible.

Studies have demonstrated that under neuroendoscopic assistance, transecting the middle meningeal artery (MMA) that supplies blood to the capsule of CSDH can effectively reduce the risk of hematoma recurrence. The underlying mechanism lies in leveraging the advantages of neuroendoscopy to transect the MMA at the lowest margin of the hematoma, thereby interrupting the blood supply to the parietal capsule of CSDH (24, 25). This may also represent a novel therapeutic direction for the management of sCSDH in the future. To date, studies also have reported that intracavitary injection of urokinase to promote septum dissolution during the removal of intrahematomal septa facilitates direct clearance under neuroendoscopy (26). This approach embodies the integration of surgical intervention with pharmaceutical therapy, serving as a promising direction for future clinical practice.

According to existing published studies, trabecular type chronic subdural hematoma (CSDH) has a significantly lower postoperative recurrence risk than other subtypes, a conclusion supported by multiple studies. Nakaguchi et al. reported a 0% postoperative recurrence rate (PR) and reoperation rate (RO) for trabecular hematomas (32 cases out of 108 lesions), with statistical significance relative to septated (*P* = 0.0003) and laminar types (27). This finding was verified by Chon et al.'s larger-sample study (420 patients), where trabecular hematomas still showed a 0% recurrence rate (30 cases), vs. 38% for septated and 21% for laminar types (28).

The extremely low recurrence rate of trabecular CSDH is linked to its natural course stage and pathophysiological features: it represents the terminal absorption/resolution phase of CSDH, has a stable internal structure with low rebleeding risk, and exhibits a mild inflammatory response (lower inflammatory cytokine levels than the high-recurrence septated type) (27, 28). Accordingly, tailored treatment strategies are recommended for preoperatively confirmed trabecular CSDH patients. Overly thorough intraoperative irrigation may be unnecessary, which helps balance surgical benefits and procedural risks (27).

In summary, neuroendoscopy has unique advantages over traditional burr hole drainage in the treatment of sCSDH, with better therapeutic effects and lower postoperative recurrence rate, which is worthy of clinical promotion. However, neuroendoscopy still has certain limitations, and it is necessary to improve the surgical method in clinical practice to enhance the immediate and long-term therapeutic effects after surgery.

In this retrospective study, considering that MRI is not a routine examination in actual clinical practice, CT was adopted as an essential preoperative examination method and an inclusion criterion. If MRI is incorporated as a preoperative examination in future prospective studies, it will enable more accurate diagnosis and treatment for patients.

## Data Availability

The original contributions presented in the study are included in the article/supplementary material, further inquiries can be directed to the corresponding author/s.
